# Impact of a tailored activity counselling intervention during inpatient rehabilitation after knee and hip arthroplasty – an explorative RCT

**DOI:** 10.1186/s12891-018-2130-7

**Published:** 2018-06-30

**Authors:** Mirko Brandes, Norman Wirsik, Hanna Niehoff, Jörg Heimsoth, Bernd Möhring

**Affiliations:** 1 Department of Prevention and Evaluation, Unit Applied Health Intervention Research, Leibniz Institute for Prevention Research and Epidemiology - BIPS GmbH, Achterstraße 30, 28359 Bremen, Germany; 2 Department of Biometry and Data Management, Unit Statistical Modelling of Primary Data, Leibniz Institute for Prevention Research and Epidemiology - BIPS GmbH, Bremen, Germany; 3 Institute of Sports Science, Oldenburg University, Oldenburg, Germany; 4 Orthopaedic Department, Rehabilitation Centre Oldenburg, Oldenburg, Germany

**Keywords:** Physical activity, Well-being, Clinical outcome, Step counting, Oxford score

## Abstract

**Background:**

The aim of the study was to improve physical activity (PA), well-being and clinical outcome after total knee and hip arthroplasty through tailored activity counselling during inpatient rehabilitation.

**Methods:**

65 patients (aged 70.4 ± 7.3 years, BMI 28.5 ± 4.3) starting inpatient rehabilitation after primary knee or hip arthroplasty due to osteoarthritis were recruited and pseudo-randomized into an intervention (IG) and a control group (CG). Twice a week, the IG was encouraged to increase their daily step count by 5%. PA, e. g. number of steps, step frequency, or active minutes, was measured by step activity monitoring. Well-being and clinical outcome were assessed using the SF-36, Oxford Knee/Hip Score and Global rating of Change. Procedures were conducted at the onset of inpatient rehabilitation, and repeated one and 6 months after inpatient rehabilitation.

**Results:**

Data sets were obtained from 49 patients (IG: *n* = 23, CG: *n* = 26). Both groups significantly increased their number of daily steps from the 1 month to the 6 months follow up after rehabilitation: CG: 9019 (95%CI: 7812, 10,226), IG: 9280 (7972, 10,588) and CG: 10921 (9571, 12,271), IG: 11326 (9862, 12,791) respectively. Additionally, well-being and clinical outcome improved significantly in both groups. No significant differences in physical activity, clinical outcome and well-being were found between the groups.

**Conclusions:**

PA counselling during inpatient rehabilitation does not improve PA, well-being and clinical outcome in patients with primary knee or hip arthroplasty in addition to the rehabilitation program. PA interventions may be more effective after the completion of the inpatient rehabilitation phase.

**Trial registration:**

DRKS DRKS00012682. Registered retrospectively on 03–07- 2017.

## Background

Around 250 million people in the world experience pain, reduced physical function, and restricted well-being due to osteoarthritis (OA) of the knee or hip. In Germany, more than 40% of the population above 60 years of age is affected by OA [1]. Therefore, knee and hip OA represents one of the most common joint disorders, and is predicted to increase further due to the aging population and obesity epidemic [2, 3]. Despite the availability of conservative treatment, joint arthroplasty is a common treatment. Clinicians as well as patients expect the treatment to reduce pain, improve lower extremity function, enable a return to a physically active lifestyle comparable to that of healthy peers, and to improve the overall well-being of patients [4, 5].

Unfortunately, due to their walking disability, patients suffering from OA are more prone to cardiovascular diseases, leading to significantly increased mortality [6]. The health risks for OA patients regarding reduced physical activity (PA), (such as a higher prevalence of type II diabetes), as well as the positive aspects of being physically active, (such as less prevalence of metabolic syndrome), were recently summarized in a vivid review by Paxton and colleagues [7]. However, several studies have demonstrated that PA after knee or hip arthroplasty is low, and that, although clinical outcomes and physical functioning appears to be good, patients do not meet current activity guidelines [8–11]. While a wide range in the amount of PA after knee and hip arthroplasty has been reported in previous research in Germany, in each study PA among patients was observed to be less compared to that of healthy peers [12–14]. Thus, the conclusion is that future research should implement PA interventions aiming to increase PA after knee and hip joint replacement. However, it is unclear at what point after joint arthroplasty PA interventions are most effective [7]. The inpatient rehabilitation is probably the earliest point at which a cost and time effective PA intervention can be delivered because hip and knee patients are then more condensed and approachable compared to immediately after surgery. In Germany, most people receive 3 weeks of rehabilitation after knee or hip replacement, starting a few days after surgery. Moreover, German patients can chose between outpatient and inpatient rehabilitation. We hypothesize that patients would benefit from additional PA interventions during inpatient rehabilitation. Therefore, the objective of the current explorative study was to implement a PA intervention after knee or hip joint replacement and to evaluate the effects of the PA interventions at one and 6 months after the inpatient rehabilitation.

## Methods

Sixty five patients (aged 70.4 ± 7.3 years, BMI 28.5 ± 4.3, 35 females, 26 knee arthroplasties) were included in the study, which was approved by the Oldenburg University ethics committee. The inclusion criterion was primary, unilateral joint replacement due to OA of the knee or hip. Exclusion criteria were revision joint replacements, additional previous joint replacement in the knee or hip on either side, any comorbidities with severe impact on the ability to perform physical activity, such as COPD, walking with crutches, and metabolic syndrome. Patients were recruited from a single rehabilitation centre in Oldenburg, which has a large catchment area in Northwest Germany. After providing written informed consent, the patients were pseudo-randomized into an intervention group, (*n* = 28), receiving the standard rehabilitation program and the individualized activity counselling, and a control group, (*n* = 37), receiving the standard rehabilitation program only. Pseudo-randomization was performed by assigning a block of 10 patients to the intervention group and having them complete the inpatient rehabilitation. After the last of the intervention group patients left the institution, a block of 10 patients was assigned to the control group and also completed the inpatient rehabilitation. This procedure was repeated alternatively until the overall number of patients was enrolled into the study. Pseudo-randomization was favoured to avoid verbal exchange between intervention and control group. The patients as well as the scientific personnel were not blinded regarding patient allocation to the IG or CG. On average, the patients started the inpatient rehabilitation 13.4 ± 5.4 days after surgery and stayed 19.4 ± 1.4 days at the centre, including weekends. The standard program of the inpatient rehabilitation included mobility and strength training, hydrotherapy, wound care, and management of everyday tasks. Physical applications were accompanied by theoretical instructions, usually provided in classes. Timing, frequency and duration of the rehabilitation components were individualized due to patient characteristics. No rehabilitation was offered on weekends.

### Study procedures

The study procedures were conducted at baseline, on the first day of arrival at the inpatient rehabilitation centre, and repeated one, and 6 months after completing the inpatient rehabilitation. A separate and quiet room in the rehabilitation centre was available for the study procedures. Physical activity was measured using the Step Activity Monitor (SAM; Step Activity Monitor 3.0, modus health, Washington, USA). The device, that has been described elsewhere [15–19], has proven excellent validity and reliability in measuring daily steps in populations with walking impairments and during inpatient treatment. According to the manufacturers’ instructions, the device was mounted to the ankle of the opposite leg of the affected limb. Gait cycles were stored at one-minute intervals. The device does not give feedback to the patients and acts as a black box. Data were downloaded using the dedicated software (StepWatch 3.4, modus health, Washington, USA) and further processed with Microsoft Excel to calculate the number of steps (twice the number of gait cycles), and movement intensity (gait cycles per minute), as well as active minutes per day with at least 60 steps/min. The average number of daily steps was considered the primary outcome of the study. The baseline measurements covered the initial days of the inpatient rehabilitation (4.2 ± 1.7 days). At one and 6 months post rehabilitation, the patients were requested to wear the device for seven consecutive days including weekends. Before each measurement, the device was adjusted to the patients’ gait characteristics (leg length and cadence).

As secondary outcomes, the valid and reliable Oxford Knee Score and the Oxford Hip Score were utilized to assess the clinical outcome in knee and hip patients, respectively [20, 21]. Patients had to answer 12 questions, each with 5 response options. The responses were valued as “4” for the best clinical option, to “0” for the worst clinical option. Thus, the outcome of the Oxford Score ranges from “0”, reflecting the worst clinical outcome, to “48”, reflecting the best clinical outcome. Well-being was estimated using the SF-36 questionnaire, a valid approach in knee and hip patients [22, 23]. The overall score as well as sub-scores for physical functioning, pain, role limitations due to physical health, and physical component score (PCS) and mental component score (MCS) were extracted. For both the clinical outcome and well-being, the data are expressed as a percentage of the maximum score, with 0% reflecting the worst clinical score or well-being, and 100% reflecting the best clinical score or well-being. Global rating of change compared to the pre-operation status was measured with a seven-point Likert-Scale ranging from 1 (“very much worse”) to 7 (“very much better”).

### Physical activity counselling

The physical activity counselling was tailored following an adaptive approach by adding + 5% in daily steps compared to the previous days. This adaptive approach has been shown to be more effective in weight loss interventions [24] and cardiology [25] than rigid recommendations, such as to ambulate 10.000 steps per day. Further, the approach was favoured by the study team because it takes the individual capacities of the patients into account. Only patients assigned to the intervention group received the counselling twice a week, preferably on a Monday and Thursday. The patients wore the SAM continuously for the entire inpatient rehabilitation period. Two students, who were familiar with the rehabilitation centre and the rehabilitation programs, were trained to be counsellors and offered the PA counselling to the IG. The counsellors downloaded the data from the device and prepared graphical visualizations of the steps taken during the previous days that they showed to the patients. Visualization included a colour figure displaying the time of day on the x-axis, and the number of steps recorded minute-by-minute on the y-axis. Furthermore, the total number of steps was displayed in the figure. Therefore, patients and counsellors were able to identify periods of walking and to estimate the number of steps taken outside the rehabilitation program, for example while walking in the park. The current data of the previous 3 or 4 days were compared to the days before that. Finally, the counsellors encouraged the patients to increase their number of daily steps by 5% over the following days, and suggested walking tasks to help meet the goal. For example: “Your average number of steps for the last days was 3000 steps. Please note that your short walk in the park counted around 300 steps. If you just add half of the short walk, you will reach the goal for the next three days.”

Because the main purpose of the counselling was to encourage the patients to make use of their restored walking capabilities, a strict adherence to increasing exactly + 5% in daily steps was less important. Consequently, regardless of whether patients increased or decreased their daily steps, they were encouraged to increase their activity with respect to the previous days by 5%. The duration of each counselling ranged from 10 to 20 min. Neither additional incentives for meeting the recommendations nor any penalties if the patients failed to increase their activity were instituted. The counselling was only provided during inpatient rehabilitation and stopped on the day of discharge from the centre.

### Statistical analysis

Parameters at onset of inpatient rehabilitation were analysed with respect to significant differences between the control group and the intervention group using an independent t-test. Differences between occasions and between intervention and control group were investigated using multi-level linear models, adjusting for age, sex and repeated measurements. This procedure allowed for the inclusion of patients measured on at least two occasions (at baseline, one or 6 months post inpatient rehabilitation). The tests were performed at a significance level of α = 0.05. All statistical analyses were performed using SAS 9.3 (SAS Institute Inc., Cary, NC, USA). We used PROC MIXED in SAS, which can handle unbalanced data and included only patients with at least 2 observations (baseline plus any follow-up examination).

## Results

**Fig. 1:**
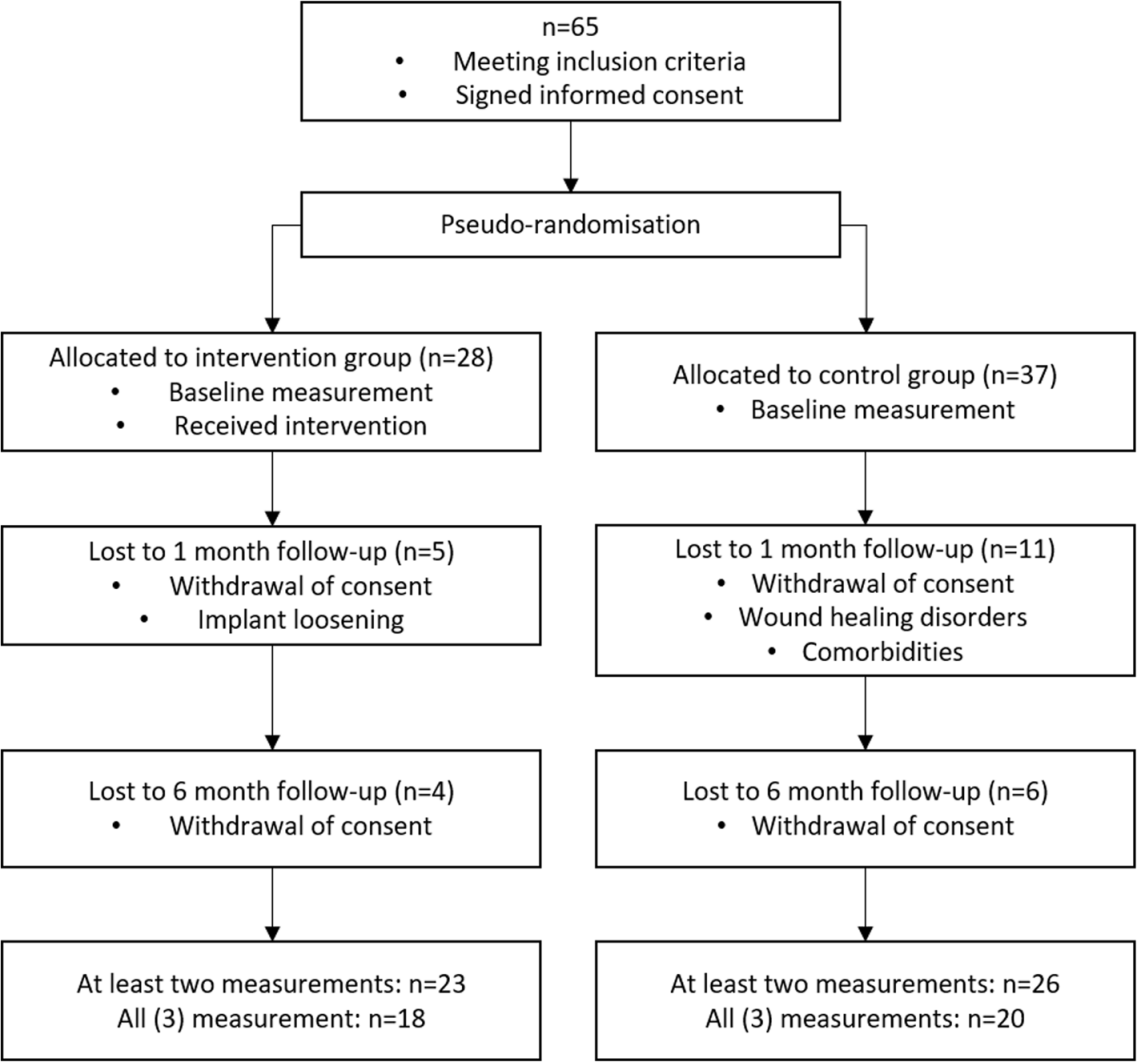
Patient flow chart

**Fig. 2:**
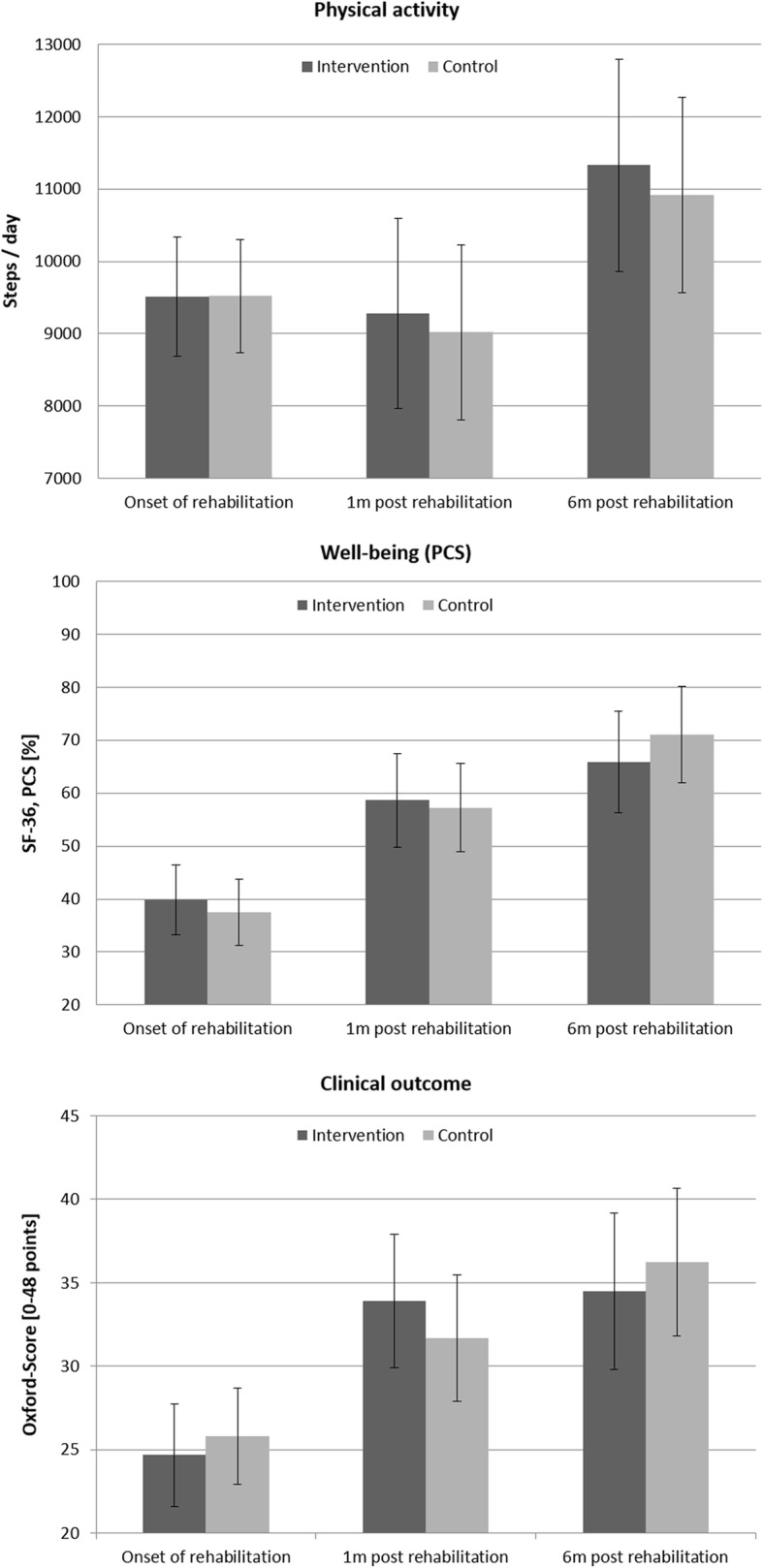
Differences between intervention (*n* = 23) and control group (*n* = 26) in physical activity, well-being (physical component score) and clinical outcome (mean, 95% CI)

Sixteen patients that signed a written informed consent at baseline dropped out of the study until the one month follow-up due to medical reasons (such as implant loosening, infections, or wound healing disorders) or withdrawal of participation (Fig. 1). Consequently, 26 patients in the control group and 23 patients in the intervention group who had at least two measurement occasions were available for statistical analysis. In anthropometric data, no differences existed between the intervention group and the control group (Table 1). With respect to the outcome data, no significant differences were observed between the intervention and the control group, neither in physical activity, nor in clinical outcome or well-being, at any time. On average, the control group and the intervention group patients wore the SAM for 4.2 and 4.4 days respectively, with a minimum of 13 h/day during the initial days of inpatient rehabilitation. At one and 6 months after inpatient rehabilitation, the control group achieved an average of 5.9 and 6.8 valid days, respectively, while the intervention group reached 6.9 and 7.0 valid days for the respective time points. The control group increased their PA from 9519 steps/day at onset to 10,921 steps/day 6 months after rehabilitation, and the intervention group from 9513 to 11,326 steps/day over the same period (Fig. 2). The average duration of inactive bouts while wearing the device decreased from 13.3 min at baseline to 10.7 min at 6 months post rehabilitation in the control group, and from 14.8 to 12.8 min in the intervention group. Both groups accumulated more minutes with active walking (> 60 steps/minute) during inpatient rehabilitation (CG = 66.6 and IG = 64.5 min), compared to 1 month (CG = 35.6 and IG = 40.3 min) and 6 months (CG = 49.6 and IG = 50.7) after rehabilitation. Further details of PA parameters are given in Table 2.

**Table 1:** Anthropometric data of control and intervention group at baseline. Data are given as mean values (95% confidence interval). IR = inpatient rehabilitation; convalescence = days between surgery and inpatient rehabilitation. Only patients considered for analysis (=at least two observations) are included

	Control group (*n* = 26)	Intervention group (*n* = 23)	p
Age (years)	69.9 (67.3, 72.5)	70.7 (68.0, 73.5)	0.61
Height (cm)	170.0 (166.3, 173.6)	171.6 (166.8, 176.4)	0.66
Weight (kg)	81.1 (76.0, 86.2)	82.2 (74.2, 90.2)	0.18
BMI	27.1 (24.1, 30.1)	27.5 (25.7, 29.3)	0.34
Hip replacements (n, %)	18 (69.2)	16 (69.6)	
Females (n, %)	14 (53.8)	12 (52.2)	
Convalescence (days)	12.3 (10.9, 13.7)	14.0 (10.9, 17.0)	0.09
Duration IR (days)	19.2 (18.6, 19.8)	19.9 (19.3, 20.5)	0.272

**Table 2:** Physical activity parameters for the control (CG) and intervention group (IG). Data are given as mean values (95% confidence interval).^1^ = *p* < 0.05 compared to 1 m post rehabilitation, ^O^ = *p* < 0.05 compared to onset inpatient rehabilitation

	Onset inpatient rehabilitation		1 month post rehabilitation		6 month post rehabilitation	
	CG (*n* = 26)	IG (*n* = 23)	CG (*n* = 26)	IG (*n* = 23)	CG (*n* = 20)	IG (*n* = 18)
Steps/day	9519 (8737, 10,301)	9513 (8692, 10,335)	9019 (7812, 10,226)	9280 (7972, 10,588)	10,921 (9571, 12,271)^1^	11,326 (9862, 12,791)^1^
Active minutes (> 0 steps/min)	295.1 (272.9, 317.2)	294.2 (271.0, 317.5)	338.6 (304.7, 372.4)^O^	328.8 (292.1, 365.5)	363.3 (328.1, 398.5)^O^	376.4 (338.3, 414.6)^1, O^
Active minutes (> 60 steps/min)	66.6 (59.2, 74.1)	64.5 (56.7, 72.3)	35.6 (27.5, 43.6)^O^	40.3 (31.6, 49.0)^O^	49.6 (39.6, 59.6)^1, O^	50.7 (39.8, 61.5)
Mean step frequency (> 60 steps/min)	40.2 (39.1, 41.4)	40.9 (39.7, 42.1)	40.4 (38.8, 42.0)	40.5 (38.8, 42.2)	41.4 (39.7, 43.1)	40.5 (38.7, 42.4)
#min with 1–20 steps/min	147.7 (131.8, 163.6)	147.7 (131.0, 164.4)	171.7 (154.5, 188.9)	162.5 (143.8, 181.2)	170.4 (156.2, 184.7)	173.4 (157.9, 188.9)^O^
#min with 21–40 steps/min	51.9 (46.1, 57.8)	52.7 (46.6, 58.8)	89.4 (79.7, 99.1)^O^	84.7 (74.2, 95.2)^O^	97.9 (87.0, 108.9)^O^	101.2 (89.4, 113.1)^1, O^
#min with 41–60 steps/min	31.9 (28.2, 35.6)	32.4 (28.5, 36.3)	43.5 (36.2, 50.8)^O^	42.7 (34.9, 50.8)	50.2 (40.9, 59.6)^O^	56.9 (46.7, 67.0)^1, O^
#min with 61–80 steps/min	38.3 (31.8, 44.7)	34.3 (27.6, 41.1)	16.8 (13.0, 20.6)^O^	18.7 (14.6, 22.9)^O^	22.9 (17.4, 28.5)^O^	25.6 (19.6, 31.7)
#min with 81–100 steps/min	18.0 (12.7, 23.2)	19.6 (14.1, 25.1)	10.1 (6.3, 13.8)	12.2 (8.1, 16.2)	13.4 (9.7, 17.1)	13.6 (9.6, 17.6)
#min with > 100 steps/min	6.6 (4.2, 8.9)	7.6 (5.1, 10.0)	6.0 (3.2, 8.9)	6.7 (3.6, 9.8)	9.9 (5.9, 14.0)	7.6 (3.3, 12.0)
Mean duration of inactive bouts (min)	13.3 (11.0, 15.7)	14.8 (12.3, 17.4)	13.3 (11.0, 15.7)	13.6 (11.1, 16.1)	10.7 (8.2, 13.2)	12.8 (10.1, 15.4)
Max. duration of inactive bouts (min)	131.8 (108.0, 155.6)	134.4 (109.1, 159.7)	124.6 (104.3, 144.9)	117.0 (95.5, 138.6)	101.0 (81.5, 120.4)	106.0 (85.5, 126.6)
Total inactive time (9 am – 6 pm)	380.5 (364.0, 397.1)	393.0 (375.3, 410.6)	371.0 (352.8, 389.3)	383.0 (363.5, 402.5)	351.2 (328.5, 373.9)	339.0 (315.1, 363.0)^1, O^
Max. mean gait frequency for 5 min	60.0 (55.7, 64.2)	58.8 (54.3, 63.3)	50.7 (46.8, 54.5)^O^	54.0 (49.9, 58.1)	56.4 (52.9, 60.0)	56.3 (52.9, 59.9)
Max. mean gait frequency for 10 min	57.2 (52.6, 61.7)	56.0 (51.2, 60.8)	45.7 (41.1, 50.3)^O^	50.4 (45.6, 55.3)	51.8 (47.6, 55.9)	52.5 (47.6, 55.9)

The clinical outcome, measured using the Oxford Score, increased significantly in both groups from baseline to 6 months post rehabilitation. Furthermore, both groups showed superior clinical outcome 6 months post rehabilitation compared to 1 month post rehabilitation (Table 3). Regarding patients’ well-being reported using the SF-36, most scores improved significantly from baseline to 6 months post rehabilitation. For both clinical score and well-being, no significant differences were observed between the control group and the intervention group at any time (Fig. 2, Table 3). Similarly, in both patient groups satisfaction with their global improvements increased significantly 1 month after rehabilitation, but not from 1 to 6 months post rehabilitation (Table 3).

**Table 3:** Clinical outcome (Oxford score) and well-being (SF-36) for the control (CG) and intervention group (IG). Data are given as mean values (95% confidence interval). PCS = physical component score; MCS = mental component score; PF = subscale physical functioning; PR = subscale for role limitations due to physical health; Pain = subscale pain; GRC = global rating of change. ^1^ = *p* < 0.05 compared to 1 m post rehabilitation, ^O^ = *p* < 0.05 compared to onset inpatient rehabilitation

		Onset inpatient rehabilitation		1 month post rehabilitation		6 month post rehabilitation	
		CG (*n* = 26)	IG (*n* = 23)	CG (*n* = 26)	IG (*n* = 23)	CG (*n* = 20)	IG (*n* = 18)
Clinical outcome		53.8 (47.8, 59.8)	51.4 (45.0, 57.8)	66.0 (58.1, 73.9)	70.6 (62.3, 78.9)^O^	75.5 (66.3, 84.7)^O^	71.8 (62.1, 81.6)^O^
Well-being	Overall	50.3 (43.3, 57.3)	54.2 (46.8, 61.6)	64.1 (56.2, 72.1)^O^	66.9 (58.6, 75.3)	74.7 (67.6, 81.9)^1, O^	72.1 (64.5, 79.7)^O^
	PCS	37.5 (31.3, 43.8)	39.8 (33.2, 46.5)	57.2 (48.9, 65.6)^O^	58.6 (49.8, 67.4)^O^	71.1 (62.0, 80.2)^1, O^	65.8 (56.2, 75.4)^O^
	MCS	63.2 (53.8, 72.6)	68.5 (58.5, 78.5)	68.7 (59.6, 77.8)	75.1 (65.3, 84.9)	77.1 (69.7, 84.4)	78.3 (70.4, 86.1)
	PF	35.4 (27.6, 43.2)	31.6 (23.4, 39.9)	58.3 (49.9, 66.8)^0^	62.9 (54.0, 71.8)^O^	74.2 (64.7, 83.7)^1, O^	67.8 (57.8, 77.8)^O^
	PR	23.4 (10.1, 36.8)	31.8 (17.6, 46.0)	39.2 (23.3, 55.1)	40.5 (23.9, 57.2)	64.1 (46.2, 82.1)^O^	59.6 (40.7, 78.6)
	Pain	25.0 (18.3, 31.7)	30.3 (23.3, 37.4)	64.0 (53.9, 74.0)^O^	64.9 (54.4, 75.5)^O^	77.1 (67.9, 86.2)^1, O^	74.0 (64.3, 83.6)^O^
GRC		4.9 (4.2, 5.5)	4.9 (4.2, 5.6)	5.8 (5.4, 6.3) ^O^	5.9 (5.4, 6.4) ^O^	6.2 (5.8, 6.6) ^O^	6.2 (5.8, 6.6) ^O^

## Discussion

The aim of the present study was to evaluate an individualized physical activity counselling intervention during inpatient rehabilitation in patients having undergone primary knee or hip arthroplasty. Six months post rehabilitation, no differences were observed between the control group and the intervention group in any measure of PA, clinical outcome or well-being.

The intervention group as well as the control group of our study showed a fairly high activity level with respect to the number of steps per day, for example compared to observations made by Tsonga et al., who reported 3518 ± 1835 steps/day for Greek elderly women treated for knee arthroplasty. However, the population investigated by Tsonga et al. included several patients with contralateral pain and/or treatments, a higher BMI and less well-being 6 months after treatment, as demonstrated by an overall SF-36 score of 62.4 ± 2.7, a PCS of 56.7 ± 2.9 and a MCS of 68.1 ± 15.3 [8]. Knee and hip patients, investigated by Harding et al., showed statistically and clinically significant improvements in the Oxford Score and well-being 6 months postoperatively compared to the baseline values before surgery. In contrast to our study findings, these patients did not improve their PA, measured by accelerometry [9]. Notably, the study by Harding et al. included patients with a higher BMI compared to our patients and 2.6 comorbidities on average. On the other hand, in another study conducted in a south-eastern region of Germany involving knee patients with a slightly higher BMI compared to our sample, Lützner et al. reported a significant improvement in daily steps from preoperative to 1 year after knee arthroplasty to 6473 ± 3654, suggesting that a higher BMI does not prohibit an increase in daily PA after rehabilitation [12]. Our own findings from a previous study including knee patients from a southern region in Germany revealed an average of 10,992 ± 3938 steps per day, and scores in well-being very similar to the current study (e. g. SF-36 overall score: 76.6 ± 16.2) [13]. Consequently, we assume that in the recent study we included a rather active population of knee and hip patients with a relatively low BMI and less comorbidities compared to other knee patient populations. We therefore presume that adoption to a physical activity intervention in these patients would be less than in knee and hip patients with a considerably lower activity. However, the PA of our patients was remarkably lower compared to the count of ~ 13,300 steps per day observed in healthy control groups of similar age investigated in two other studies from Germany [12, 14]. Thus, the patients in our study ambulated less than their healthy peers at 6 months post operatively, implying that they might potentially benefit from PA interventions to achieve equity with their peers.

We did not observe any effects of the individualized activity counselling during inpatient rehabilitation in the intervention group. Although patients were satisfied with talking to the counsellor every 3 days and reflecting and discussing their PA, they were not able to systematically increase their daily PA. The daily PA measured during the inpatient rehabilitation showed rather random movement patterns, with a large day-to-day variability (data not shown). Therefore, we assume that the patients were not able to focus on their daily PA due to the rehabilitation program demands. Although patients agreed to aim for reaching 5% more steps than performed in the previous days, they often reported a need to recover from the standard rehabilitation program before they could start on their PA aims. This response is understandable as it is known that lack of energy is a common barrier to undertaking physical activity [26, 27]. Thus, we conclude that the PA counselling had no impact on objectively measured PA.

The decline in PA in contrast to an increase in clinical outcome and well-being from presurgery status to 1 to 3 months after rehabilitation, followed by a further increase in PA, clinical outcome and well-being from 3 to 6 months after rehabilitation has been observed in previous studies [8, 13]. However, in our study the baseline PA values do not represent presurgery status. Instead, the number of steps taken was influenced by the schedules and the program of the inpatient rehabilitation and does not represent habitual daily steps. Nevertheless, both the control group as well as the intervention group ambulated less when they returned home, compared to during the inpatient rehabilitation. We assume that after joint arthroplasty patients have to rearrange their daily life when returning home, and that this time is characterized by less continuous activity as reflected by the active minutes with a minimum step count of 60 steps per minute. Instead, patients show non-continuous walking patters as reflected by the active minutes without a minimum step count per minute. But 6 months after rehabilitation, patients were able to increase their overall daily PA. Both groups reduced the duration of inactive bouts and instead accumulated more active minutes as detected by the SAM. These active minutes are distributed well over the entire spectrum of walking frequency, but were pronounced in the moderate walking frequencies from 21 to 100 steps/min. However, the patients did not achieve the PA level of their healthy peers even 6 months after rehabilitation.

The clinical outcome and well-being significantly improved from the onset of inpatient rehabilitation until the 6 months follow up. Interestingly, the control group showed lower values 1 month after rehabilitation, but exceeded the clinical outcome and well-being of the intervention group 6 months after rehabilitation, except for the mental component score.

Some limitations concerning our study have to be addressed. Unfortunately, we lost 16 patients after successful baseline measurement. The remaining number of 49 patients included in our study might not have enough power to disclose any significant differences between the control and intervention group and between hip and knee patients. There was an additional medical reason why we chose the step activity monitor as the intervention tool, and not providing continuous feedback to the patients. Our intention was to avoid overexertion induced by a lower step count as well as inter-individual comparisons between patients. We are aware that this approach is rather conservative, and we hypothesize that, at least in some patients, a pedometer with continuous feedback would have increased their PA.

The fact that we did not have presurgery data for our patients limits our findings. The rehabilitation centre of Oldenburg, where the intervention took place, receives patients from a catchment area covering a large proportion of Northwest Germany. Furthermore, as most patients in Germany can choose the rehabilitation centre they want to go to, it is not possible to get prior information on which patients would chose the Oldenburg Centre for inpatient rehabilitation. Thus, we could not relate the physical activity of the patients included in our study to their pre-surgical physical activity. Moreover, our inclusion criteria led to a rather fit sample of knee patients. Consequently, our findings should be transferred to other patient populations, e. g. with severe comorbidities or higher BMI, with caution. Potentially, adoptions of PA interventions are expected to be higher when baseline PA level is lower. Therefore, we recommend that our study be repeated in different patient populations. We were also not able to measure daily PA during inpatient rehabilitation in the control groups, thus we have no information on whether the counselling in the intervention group altered PA during inpatient rehabilitation.

In this study, we utilized a step activity monitor blinded to the patients as it does not show the recorded number of steps to the patient. On the one hand, this limits the possibilities of the patients to check if they have reached the intended number of steps. On the other hand, patients were not able to compare their daily step counts with each other, reducing their chances of challenging each other to perform more steps. This could potentially lead to excessive demands or overloads being made on the joint treated.

## Conclusions

The results of this study indicate that physical activity counselling, based on the daily number of steps during inpatient rehabilitation, does not lead to better outcomes after discharge. Consequently, we suggest that interventions aiming to improve daily PA should not be implemented during inpatient treatment in addition to the standard rehabilitation program because the physical capacities of knee and hip patients are fully claimed by the rehabilitation program. Due to the difference in PA between patients and their healthy peers, we recommend that the implementation of PA counselling during the period following inpatient rehabilitation, when patients resume their daily life at home, be further examined.
